# COVID-19 Vaccination Rollout: Aspects of Acceptability in South Africa

**DOI:** 10.3390/vaccines10091379

**Published:** 2022-08-24

**Authors:** Bent Steenberg, Nellie Myburgh, Andile Sokani, Nonhlanhla Ngwenya, Portia Mutevedzi, Shabir A. Madhi

**Affiliations:** 1 South African Medical Research Council Vaccines and Infectious Diseases Analytics Research Unit, Faculty of Health Sciences, University of the Witwatersrand, Parktown 2193, South Africa; 2 African Leadership in Vaccinology Expertise, Faculty of Health Sciences, University of the Witwatersrand, Parktown 2193, South Africa

**Keywords:** South Africa, COVID-19, vaccine acceptancy, vaccine hesitancy, vaccine denial

## Abstract

Unprecedented in scale, immense COVID-19 immunization programs have been rolled out globally. This article explores aspects of hypothetical vaccine acceptability in Soweto, South Africa, shortly before such vaccines became available. Whereas hypothetical acceptance was normative, this has not translated into uptake today, which remains concerningly low in South Africa, especially in Soweto. For that reason, we mobilize anthropological concepts to analyze acceptance, hesitancy, and denial to gauge public proclivity to inoculate. We found that COVID-19′s haphazard mediatization generated a ‘field of suspicion’ towards authorities and vaccination, which, amplified by dis- and misinformation, fostered othering, hesitancy, and denialism considerably. Further, we demonstrate that stated intent to immunize cannot be used to predict outcome. It remains paramount during vaccination rollouts to unveil and address aspects detrimental to vaccine confidence and selectivity, especially in lower-income groups for underlying context-specific cultural, spiritual, historical, and socioeconomic reasons. Appropriate mediazation alongside a debunking of counterfactual claims is crucial in driving forward immunization.

## 1. Introduction

Coronavirus Disease 2019 (COVID-19) is a Severe Acute Respiratory Syndrome (SARS), which has so far claimed more than six million lives worldwide (July 2022). Since its initial discovery in China in December 2019, it spread across the globe causing grinding lockdowns and economic disruption exacerbated by war in Europe and climate change [1]. These unprecedented financial downturns have reversed gains in terms of health indicators. Preventive measures have included social (meaning physical) distancing, the closing of schools and businesses, and the wearing of face masks in public. Whereas this may have flattened epidemic curves, COVID-19 resurged as economies around the world gradually reopened.

Safe and effective vaccines have been developed with some still undergoing clinical trials [2]. In December 2020, for instance, there were “61 COVID-19 vaccine candidates awaiting clinical evaluation and 172 candidate vaccines in preclinical evaluation” [3]. Immunization is a vital public health achievement and has substantially reduced illness and significantly decreased mortality and disability [4]. That said, the success of any vaccination program depends on public acceptance [5]. Acceptability refers to how well interventions are understood and received by target populations, and the extent to which these meet their needs [6]. Presently, there are lacunae in the existing literature’s comprehension of how stated intention to inoculate, meaning hypothetical acceptability, translates into (or can be used to predict) uptake and outcome—an evidence gap this article aims to bridge.

Acceptability is one of the quality components in healthcare, apart from efficiency, optimality, legitimacy, and equity. It is defined as conformity to the wishes, desires, and expectations of patients and responsible members of their families [7].

Governments worldwide provide free inoculation to mitigate the impact of COVID-19 by way of reducing morbidity and easing pressure on strained healthcare infrastructure. However, the public’s readiness and willingness to accept vaccination is not fully understood and studies have gauged hesitancy and denial. Further, acceptance is tethered to location and culture, leading some to question the ‘pan’ in pandemic and suggest that “different versions of a pandemic can co-exist” in what Marsland has referred to as polydemics due to this perceptional plurality [8]. In countries such as the UK, France, Germany, Italy, Holland, and the USA, COVID-19 vaccination acceptance was relatively high as rollouts launched: around 60 percent or above [9]. However, despite widespread acceptance, many communities still harbor deep-rooted doubts and concerns about vaccines, causing both hesitancy and denialism [10].

Reasons for indecisiveness are multiple and interwoven. For instance, hesitancy may stem from historical mistrust in medical establishments or in government’s authority. This is then influenced by friends, family, and colleagues, and further inflected by media, ranging from local social platforms to global satellite networks [11]. For some, vaccination may not have seemed urgent—vaccines were still undergoing trials and it might not have seemed like the ‘right time’ to immunize. Meanwhile, logistical discrepancies can lead to lack of access to vaccines [11]. According to the World Health Organization, ‘vaccine hesitancy’ refers to: […] delay in acceptance or denial of vaccines despite availability of vaccination services. Vaccine hesitancy is complex and context-specific varying across time, place, and vaccines. It includes factors such as complacency, convenience, and confidence [3].

To characterize inoculation as one of the foremost successes in public health would not be an overstatement. However, firm vaccine deniers (or ‘anti-vaxxers’) do not share this view and perceive vaccines as unsafe and unnecessary. One central reason for this is the mediatization of counterfactual claims—unfounded misinformation about vaccines, vaccine safety, or even the origins of COVID-19 as manmade or manufactured to control or decimate populations or races—so-called conspiracy theories.

In the following, the contributing authors mobilize the anthropological concepts of mediatization, fields of suspicion, othering, and counterfactual claims to better understand human behavior in relation to hesitancy and denialism. Studies have identified denial determinants as fear of side effects, failing recommendation to vaccinate by trusted individuals or healthcare providers, adverse attitudes towards healthcare services, preference for traditional phytomedicines, cultural praxis, or religious beliefs [12]. So far, with respect to COVID-19, reasons for hesitancy include its novelty and the vaccines’ unusually short production time span. To this, one might add negative past experiences with healthcare or lack of awareness of inoculation. Also, simply, the fear of pain at immunization and fear of needles are also drivers of denial [13].

Vaccine hesitancy and denial are major stumbling blocks to the successfulness of vaccination rollouts. Hence, an anthropological weighing of a community’s acceptancy—as well as identifying reasons underpinning hesitancy and denialism—may accelerate uptake when translated into policy and practice. In the following, we explore such aspects of acceptability in South Africa where uptake is currently low; less than 32 percent of the population in July 2022.

## 2. Methods

An exploratory qualitative study was undertaken in Soweto and Thembelihle, South Africa, in August 2020. Here, to understand factors contributing to hesitancy or denial, we explored these communities’ acceptability towards COVID-19 immunization, which were being developed and not yet available at the time. An ethnographic approach was adopted to examine perceptions, perspectives, and attitudes towards vaccine-related issues, particularly those giving rise to anxiety [14]. Fieldwork was carried out in Soweto and Thembelihle, specifically in the township clusters of Braamfischer, Emndeni, Mapetla, Meadowlands’ zones four and five, Mofolo North, Phiri, Senaone, and Thulani townships. Soweto and Thembelihle form part of the Johannesburg Metropolis in Gauteng and has a population of approximately 1.7 million consisting predominantly of low to middle-income Black households.

Informants were sampled to gather data representative and illustrative of the communities’ acceptancy of COVID-19. Ethnographic fieldwork involved informal interviewing in a casual ambience and elaborate fieldnotes [15]. All interviewees were briefed and signed informed consent forms beforehand. Qualitative data were collected during focus group discussions (11) and key informant in-depth interviews (5) from 66 adult community members (21 men; 45 women; mean age 38; range 18–65 years).

With the assistance of a local community advisory board, these were selected from the unemployed (25), employed low-income earners (15), and self-employed small business owners (13), pensioners (8), and caregivers of infants or children. This included occupations such as retail shop workers, hair stylists, dressmakers, schoolchildren, transport drivers, cleaners, security guards, domestic workers, delivery boys, restaurant waiters, and families on social grants: pensioners, child-headed families, caretakers, or people living with HIV or disabilities.

All interviews and focus group discussions were digitally recorded and then transcribed verbatim by research assistants. These data were then coded and analyzed using computer-assisted qualitative data analysis software (NVivo12). From the analysis, overarching themes were identified regarding COVID-19 acceptability. The Human Research Ethics Committee of the University of the Witwatersrand approved the protocol, clearance no. 201003.

## 3. Findings

While more than half of our informants seemed reasonably informed about vaccination in general, we also found what is perhaps best characterized as perplexing confoundment regarding knowledge, opinions, and attitudes towards COVID-19 and immunization. In our analysis, we argue that a ‘field of suspicion’ towards the virus, vaccination, and health authorities was amplified at the time, partly due to the nature of COVID-19′s mediatization combined with the site-specific cultural and historical contexts of Soweto and Thembalihle. This was then further aggravated by a social mechanism we refer to as ‘othering’, and, finally, exacerbated by circulating counterfactual claims and conspiracy theories. As we demonstrate in the following, this amplification of doubt and incertitude came to underpin and fuel aspects of vaccine hesitancy and denialism significantly. We conclude that stated intention to immunize does not predict outcome.

## 4. Acceptance

Vaccine acceptance relies on public understanding and reception of a vertical intervention. It is prudent to commence conversations with community members at an early stage to understand factors that may affect acceptability, and then develop means to address and counter those preoccupations. Once an effective vaccine is available, it should be rolled out in a timely fashion and be made easily accessible to the community. This requires healthcare system capacity and communication strategies to build trust and increase acceptance.

Three different surveys reporting on acceptance levels in South Africa found these to be 76 percent, 67 percent, and 82 percent, respectively [16,17]. More recently, a review of surveys investigating COVID-19 vaccine acceptability conducted in South Africa from February 2020 to March 2021 revealed its “inherently social nature” in that it is influenced by culture, race, politics, trust, and geographical location—findings we echo and explore further here [18].

Similar surveys from elsewhere in the world have consistently tabulated lower levels: 60 percent in the USA, 56 percent in England, and only 35 percent in China [9,19,20,21]. Global surveys reported an average vaccine acceptancy rate of about 70 percent with countries with more government control having higher acceptance than those with less [16]. However, common contextual factors associated with decreased vaccine acceptability in high-income countries alone do not differ markedly from factors identified elsewhere. For instance, common factors in high-income countries are (similarly) “not being of white ethnicity and lower education” and “beliefs that vaccines are not safe/effective and increased concerns about rapid development of COVID-19 vaccines” [22].

Our study is among the first to have collected data on vaccine acceptancy in low-income communities at a time when the number of COVID-19 cases was increasing rapidly in South Africa. We found that 36 informants (55 percent) were willing to immunize were a vaccine to become available, 22 (34 percent) were not, and 8 (11 percent) were hesitant. Hence, we distinguish between three aspects of acceptability: acceptance, hesitancy, and denial. For example, shrugging, one woman was opposed while another conceded:

*Well, yes, I refuse because I want to survive.*

*I’ll be the first to take it. Yeah, anything to live, as long as I don’t have to keep living with this bloody mask.*

One youth expressed solidaric reasons for embracing the vaccine:

*I think it’ll help many people and older people are the ones who will benefit the most.*

There was widespread consensus regarding getting ‘vaxed’ to assist high risk groups in the community. For instance:

*I don’t have a problem as long I know it’ll benefit other people.*

*I wouldn’t have a problem with that.*

Parents often indicated that due to a desire to go back to normal, pre-lockdown life—meaning getting rid of masks and for children to resume school—they would accept inoculation. Those who based their decision to immunize on safety and survival seemed confident that the vaccine could save their lives and the lives of loved ones. The main reason for vaccinating was thus to protect oneself *and* others from the virus. In fact, several informants wanted to immunize precisely because it would help others, especially the old and the vulnerable. Our data also suggested that some informants’ reasons for vaccinating stemmed from traumas of bereavement due to COVID-19. Namely, three informants seemed distressed as they discussed it.

I think the vaccine will be an answer to Coronavirus. Yeah, it will, and I also think about the community, because most who have seen people dying from Coronavirus, they will run for the vaccine.

I believe that when the vaccine comes, everyone will want to be vaccinated. No one wants to die. We do want to be vaccinated because we’re afraid of dying. 

We have all heard that there is a vaccine, and we want its development to happen. Once we are all vaccinated, the gravesites won’t be full before time anymore.

Based on these statements, most parents and guardians reported that they would accept a COVID-19 vaccine for themselves and their loved ones. Other informants responded that their decision to vaccinate or not would be influenced by the fact that two vaccine trials were being carried out locally in South Africa.

For me, I would vaccinate, mainly because it’s made right here. Meaning that you have a background understanding of the community’s needs as well as its fears. I would support it because it’s made here in South Africa.

People generally attribute importance to news or events proportionate with their proximity to home or the ‘local’. In much the same way, local conditions “shape perceptions of public health interventions and [also] how conspiracy theories come into play” [23]. In short, it is the local that shapes “people’s reception of, and by extension compliance with, governmental public health emergency efforts” [20]. We may then reasonably ask, firstly, how local public narratives and ‘knowledge’ about COVID-19 emerge and evolve and how this knowledge is disseminated in the community—which brings us to discuss mediatization and fields of suspicion.

Initially, we should establish that deep inequalities in South African society offer differential access to communication technologies—smartphones, data affordability, et cetera—which may make vertical information dissemination particularly challenging in townships like Soweto. Briggs [24] posits that “we do not live in societies with media but in mediated societies where images of self and society are shaped by the media.” In other words, communication shapes the public in that “public discourses help create the publics they purport to address, as people position themselves in relationship to circulating messages or just let them go.” [24].

It then becomes imperative to ask “how access to production and reception of authoritative knowledge about disease is distributed” [24]. Ideally, experts, in the present case public health experts and policymakers, would become the “primary definers of emerging narratives about the pandemic” [24]. Instead, on the contrary, “fraught discussions ensued regarding COVID-19 cover-ups, pandemic geopolitics, bot and humanly driven disinformation floods on social media, and reporting bias in the press” [23].

This morphed into an emergence of what Fairhead et al. called a ‘field of suspicion’ (or of ‘uncertainty’) [25]. Essentially, decisions to immunize are “taken in a field of uncertainty and speculation in which wider confidence or worries are relevant” [25]. Thus, as Sobo points out, rampant pandemics (or polydemics) can engender “conspiratorial interpretations where a history of health-related abuses exists and obvious disparities in who gets sick will feed suspicion”, which would also be an ominous, yet pertinent description of wider South African society as a whole, but especially of Soweto [26]. Marsland, illustrates that this field of suspicion (or ‘not knowing’) reflects “a combination of secrecy, uncertainty, and skepticism in related medical knowledge” [8]. In fact, fields of suspicion are significantly amplified by conflicting messages from authorities:

The uncertainty regarding specific origins [of COVID-19], successful cures, prevention measures, and risk for transmission expressed within the scientific community is exacerbated in the public mind by conflicting messages from different health authorities. [26].

In summary, the multidirectional mediatization of COVID-19 produced a field of suspicion in the community—as we learned from our interactions with Sowetans—in which almost half of our informants were swayed from acceptance into hesitancy or denialism.

By now, we have a clearer picture of the main reasons for acceptance: safety and survival, the desire to return to ‘normal life’, and the notion of not having to wear a mask, which, at the time, was a novel nuisance. Finally, solidarity and the belief that a vaccine might aid other people is a contributing factor alongside hearing from confidants whose opinions are trusted—or who have lost a loved one to COVID-19.

## 5. Hesitancy

Vaccine hesitancy is the inability to decide whether to immunize. It is a wavering indecisiveness during which individuals attempt to make sense of conflicting arguments for and against in an ambit of incertitude and angst circulated by peers and through media channels and platforms. Hesitancy stems chiefly from conversations with friends, family members, colleagues, and inputs from COVID-19′s contradictory and haphazard mediatization despite the (here hypothetical) availability of vaccination services. Furthermore, individuals may come to believe in counter factual claims or be (understandably) confounded by them. They may also simply lack knowledge about what vaccines are or how they work—causing this wavering within a now amplified field of suspicion [25].

We found concerning levels of vaccine hesitancy (or indecisiveness) among low-income earners in Soweto. This hesitation was swayed by several intertwined, local factors ranging from doubt in vaccine efficacy to cultural and spiritual praxis. About one in every ten of our informants were hesitant, stating that more adequate information to validate the efficacy of the vaccine would increase their likelihood of accepting it. Others thought it prudent to wait and see what results it might produce in others and insisted on certain safety measures before agreeing to immunize.

I can take it after some time when I have seen how it works. I won’t be part of the first people to get it.

Likewise, in a study on acceptability and willingness to pay for hypothetical Ebola immunization in Nigeria, most of those hesitating affirmed that they would only accept a vaccine after observing its outcomes in others [27]. Meanwhile, with respect to information and knowledge:

These things (vaccines) aren’t one hundred percent right. These people are playing with our lives, they should take a donkey or a cow and test it first.

There are clear misunderstandings here and a lack of elementary comprehension of how clinical trials are orchestrated. Similarly, some participants laid out the conditions under which they would vaccinate against COVID-19 as “testing positive for it first” and so apparently did not grasp even the basics of immunization. Some stated that they would only “take the vaccine” were they to be encouraged to do so by a superior at work. One aging man believed that inoculation would be a “blood transfusion” and suggested diabolical influences:

It also depends on how it’s done, because if it’s a blood transfusion, that’s bad. […] This thing is demonic [COVID-19].

Vaccinologists have suggested that misinformation regarding vaccines, and poor knowledge of them, may induce anxiety and perceived (fields of) uncertainty, resulting in an overestimation of potential side effects [28]. Again, perceptions recounted by low-income earners seemed tilted by people whose opinion they trusted. Compared to other groups, hesitant groups do take into consideration authoritative advice from health or government officials in their vaccine decision making.

Qiao and colleagues proposed that to reduce concerns about the safety of COVID-19 vaccines, evidence-based health communication “should address misinformation about vaccines (e.g., ‘fact check’) and deliver vaccine knowledge using population-appropriate languages” [20]. In Soweto, however, where communications have primarily been biomedical and clinical, what is a ‘population-appropriate language’ in townships where the overwhelming majority frequent *sangomas* (traditional healers) and have an arduous history of exclusion and marginalization brought down upon them by their own state?

Back in 2020, a global survey of COVID-19 acceptability demonstrated that levels of vaccine hesitancy globally were high and probably rising [16]. Today, there is growing concern about rising vaccine hesitancy everywhere. Olson and colleagues demonstrated that in many countries vaccine hesitancy and misinformation pose a huge obstacle in achieving coverage and community immunity [29]. In our analysis, we too found misinformation to constitute a major hurdle. Again, this accentuates the importance for public health officials to curtail hesitancy by improving vaccine literacy [30].

According to Lazarus et al., the speed by which misinformation spreads through multiple channels has a detrimental effect on acceptancy worldwide. Several participants aired counterfactual claims and elaborated endlessly on (so-called) conspiracy theories to justify resistance towards immunization [16].

They put laptop [chips] in the body.

We will be getting shots and be controlled by 5G [5th generation mobile network].

Social media informs decisions. Due to COVID-19′s mediatization, some Sowetans hesitated or denied inoculation, they said, because of unfounded and undocumented rumors that people who had volunteered for vaccine trails had subsequently passed away. On another occasion, a “doctor” was rumored to be offering money to subjects willing to “participate in tests”. These and similar accounts are indicative of an evolving perception of the virus and vaccination that reflects the deep unreliability of developing narratives and discourses across different media platforms.

This is especially true of social media gossip and surely worsened by a paucity of critical thinking and conflicting messages from authorities, which brings on hesitation and creates doubt that was long ago “shown as a logical response to informational discrepancies […].” [26]. Doubt, in turn, creates discomfort. However, problematically, an “erosion of trust in authoritative and scientific knowledge does not extinguish the ‘will to truth’”, but rather “opens a space for alternative forms of knowledge” [23]. 

This ‘will to truth’ then seeks its ‘alternative knowledge’ and finds it in counterfactual claims and conspiracy theories, which have abounded throughout the pandemic and still do. Conspiracy theories have certain common denominators, namely that they always imply nefarious intent, that ‘something must be wrong’, and that nothing occurs by accident. Furthermore, they can be self-contradictory and entirely override suspicion in themselves in that they are immune to any form of evidence and inherently ‘self-sealing’. Meanwhile, conspiracy theorists perceive and present as victims of organized persecution, and, at the same time, “see themselves as brave antagonists taking on the villainous conspirators.” [31].

COVID-19 ‘escaped’ from a Chinese laboratory, the 5G network is causing it, Bill Gates was scapegoated, it was created as a biological weapon, the US military deliberately imported it into China, and, somehow, genetically modified crops caused COVID-19, while the virus does not actually exist, and the pandemic is manipulated by a ‘deep state’, because COVID-19 is a plot brought on by ‘Big Pharma’, and its deaths rates are purposely inflated.

In this light, it is perhaps not entirely incomprehensible that ordinary men and women might lose themselves in this maddening maze of disinformation and choose to defer their decision to immunize in an uncomfortable ambit of nagging doubt and incertitude.

In a similar vein, a workman in dungarees related another incident in which individuals or institutions entrusted with the provision of face masks to protect the public had in fact infected these same masks with Coronavirus deliberately.

They already gave us these masks that got us infected and now they want to finish us? No!

Key here is the word ‘they’ (as opposed to an ‘us’), which is indicative of a social mechanism anthropologists have referred to as (transformative) ‘othering’ [32,33]. In profound mistrust bordering on paranoia, whomever ‘they’ may be, they remain nameless, distant, shadowy figures that “want to finish us”. Even if seen as either a self-defensive mechanism or one affirming social identity and belonging, according to this logic, ‘we’, the people of the community, are blameless victims as it was plainly ‘them’, the others, the Whites, the rich, the Chinese, the shadow state, Bill Gates, or perhaps demons that deliberately unleashed this evil ‘Kung-Flu’ upon ‘us’.

Could it be that perhaps it was biological nature? Perhaps, but communities, it seems, must have their culprits and scapegoats; never us, always them, the mekwerekwere, the foreigner, the other. According to Onoma, “the scapegoating of certain populations during disease outbreaks is very common [and] outbursts usually target noncitizens, foreigners, and those generally cast as ‘other’” [34]. In short, it is then a process of constructing these others as public health dangers. Sobo notes that “such opposition intensifies misunderstanding, proliferating false binaries of ‘us vs. them’ and ‘truth vs. fiction’”, which further amplifies our field of suspicion [26].

While informants highlighted risks related to the uncertainty of novel vaccine trials, one grandmother pointed out that monetary compensation offered to clinical trial volunteers was not worth the risk. Another concurred.

The testing is starting out [trials]. So, when they test you, what if you die? Our system can’t handle it [immunization]. It would’ve been better if we were used to it [vaccine trials], but now it will be difficult unless people get millions for it. We are scared of death.

There are people now who have funding in Soweto to test vaccines and those vaccines are going to make people sick. How sure will we be that the vaccine is here and that it is the right one? If you’re not, then you have to withdraw from participating [in trials].

Out in the vast, sprawling townships, people oftentimes questioned conditionalities of immunization and thus demonstrated hesitancy and perceived incertitude. A filling station attendant gave it some thought and then proposed that:

*I would be happy if a vaccine were available, but with what conditions will it come? Is it for free or do we have to pay for it? There’s no way they can say that it’s for free because there’re many of us, and if they say we must pay, how much will it be, and who can afford it, because obviously, if it’s pricy, then it’ll only be available for rich people.*

Questioning the integrity of the information that informants had so far received about inoculation, the intentions of stakeholders advocating vaccination came under suspicion. A car washer swiped her long, braided hair and asked:

*Why do they want to try the vaccine when people are recovering already?*

*Another informant doubted the reliability of the vaccine seeing as how it was being sourced from foreign countries.*

*Why should we believe a vaccine that’s mixed [manufactured] outside our country?*

Such statements illustrate profound suspicion of the trustworthiness of foreign institutions (and foreigners in general) as well as any local entities carrying out their interventions. Again, there is ‘othering’. Again, we see poor knowledge, mistrust, uncertainty, and doubt engendered by and further engendering misinformation, menacing rumors, and counterfactual claims in a downward spiraling self-perpetuating circle of half-truths and debilitating distortion.

Vaccine hesitancy has been defined in public health literature as caused by a combination of beliefs, attitudes, and behaviors in laypeople in relation to immunization. The choice between vaccine acceptance and denialism is determined by individuals’ perceptions, preferences, and motivations as well as a polydemic’s prevalence in society.

Vaccine hesitancy is an attribute ascribed to a large and heterogeneous category that regroups people who share varying degrees and motives of indecision and who hold an intermediate position along a continuum, ranging from full support for vaccination to strong opposition to any vaccine [35].

In what constitutes the perceptual pluraversality of COVID-19, cultural backgrounds, beliefs, and attitudes do influence laypeople’s decision whether to ‘vax’ or not, and, in turn, “vaccine perceptions are shaped by complex socio-political and historical factors” [17]. As such, vaccine hesitancy is a decision-making process informed by individuals’ commitment to health, risk, culture, and their level of confidence in health authorities and medicine [35]. Those hesitating to inoculate generally lack vaccine confidence, meaning that they distrust vaccines overall as ‘safe’ or mistrust healthcare authorities and political decision-makers, and, more often than not, Sowetans seriously doubted the intentions of the conspicuously corrupt latter. Hence, the mere availability of a vaccine does not necessarily imply uptake.

In Soweto and Thembelihle, it soon came clear that more adequate information is required to curb hesitancy and denial. This includes information about vaccine safety, effectiveness, and trial processes as well as appropriate language to communicate with specific groups, such as the elderly, parents, or youths. In summary, the following fueled hesitancy in particular: a low visibility of successful outcomes of inoculation, mistrust in the (ill) intentions of government authorities, suspicion of vaccines manufactured abroad (by ‘others’), doubt in safety measures and the trustworthiness of entities developing vaccines, a lack of transparency and adequate information, anxiety induced by (dis)information circulating in media, especially social media, and doubt in the actual severity of COVID-19 infection (or its origins for that matter): the polydemic’s perceptual pandemonium.

## 6. Denialism

Vaccine denialists, including COVID-19 conspiracy theorists—or in more militant jargon: ‘anti-vaxxers’—reject immunization even when available and despite its benefits clearly outweighing its miniscule risks [36]. Denialism, just as with hesitancy, is influenced by a host of factors such as confoundment, contradictory messages, disinformation or outright untruths, or previous challenging experiences with healthcare services resulting in reluctance to procure them [37]. Several of our informants were firmly opposed to inoculation, because of incertitudes about efficacy or safety. As one healthcare worker recalled with trepidation:

*People from Braamfischer chased us out when we went there. They thought we were going to inject them with the vaccine until we explained to them what we’re about.*

One gentleman shared with us his lack of confidence in the vaccine, indicating a strong inclination towards alternative protective measures:

*No, I was not going to take it, as I did not even test for COVID-19, but I do take prevention methods all the time. I believe more in the African way of doing things.*

Phrases such as ‘the African way of doing things’, and many similar ones recorded in Soweto and Thembelihle, were oftentimes used interchangeably with notions of traditional medicine, usually in the form of herbs or ancestral worship. One elderly man bemoaned government’s disregard for non-conventional medicinal solutions while others denied inoculation unequivocally:

*Our government would rather have these vaccines tested on us rather than rely on our natural herbs, so you can see that our government is really turning its back on us.*

*I would never take it!*

*I would never [vaccinate]!*

That first statement brightly elucidates some of the cornerstones of denialism, namely, suspiciousness of government’s benign intentions, belief in traditional phytomedicines (medical pluralism), and mistrust in authorities blended in with a foreboding sense of abandonment: “our government is really turning its back on us”. If one so feels and thinks, is it any wonder that one then vehemently shuns and denies immunization? In a study from Vietnam, reasons for vaccine denialism were reported as doubts and concerns about the efficacy and safety of the vaccine, rather than believing it to be unnecessary, or simply not having time to go for it [21]. This was also true in Soweto and consistent with reports from the 2009 H1N1 pandemic, when people were mostly concerned about side effects [21].

Although over 90 percent of the respondents stated that they would accept the COVID-19 vaccine when available, almost 50 percent of these people wanted to delay the vaccination until it was confirmed safe. People were unlikely to accept the COVID-19 vaccine [because of concerns over] risks, safety, and side effects of vaccination. One study reported that over 95 percent of the respondents had concerns about the efficacy, effectiveness, and safety of the COVID-19 vaccine [21].

Many informants signaled that they were not willing to immunize, because the community did not believe that it would be beneficial. In addition, impoverished people are obviously less likely to inoculate if this implies financial expenditures—and it does because of transport costs and time spent away from income generating activities. Just as with hesitancy, vaccine denial is pluraversal and comprised of compounded factors. Based on the statements presented above, it comes to light that multiple approaches are required to address multiple aspects of acceptability. Vaccine denialists (and hesitators) also seemed concerned about the negative impact immunization might have on human bodies in terms of side effects and suspected, but undocumented long-term consequences: “you can turn into a monkey”, said one girl. Such sentiments are clearly detrimental to vaccine acceptability and these concerns may become particularly salient when a vaccine is new and rapidly developed [38,39].

In summary, vaccine denialism is fostered most prominently by financial costs, uneven access, lack of confidence in successful outcomes, faith in traditional phytomedicine, lack of reassuring information, vaccine-skepticism stemming from trusted companions and confounding mediatization, alongside whispers of counterfactual claims and conspiratorial schemes.

## 7. Conclusions

Despite relatively high hypothetical vaccination acceptance in the communities, uptake can be much improved—although intent may be present, more often than not, it does not translate into action. In other words, stated intention to immunize does not predict outcome. We saw 55 percent of our informants (and 75 percent on average in national surveys) accept inoculation putatively, whereas in Soweto today, only around 20 percent have finished their immunization, suggesting that little less than two thirds of those who said they would immunize did not. Initially, information about how vaccines were developed and tested, including their safety and efficacy, could have been communicated in a sounder fashion. Instead, contradictory messages and counterfactual claims abounded in COVID-19′s mediatization. This, in tandem with widespread absence of even basic notions, comprehension, and knowledge about how vaccine trials and inoculation function, further amplified a field of suspicion towards the virus, immunization, and health authorities. Add to this the (much abbreviated) list of COVID-19 conspiracy theories presented in the above, and the uncomfortable doubt and increasing uncertainty become more understandable. Added to this confusion is an inherent human fear of death in a population that oftentimes prefers traditional African remedies over modern biomedicine. Throw into this blend an under-capacitated government marred by corruption, which has given its people little reason to trust it, while on the contrary, historically, it has given them plenty of reason not to. Lastly, an ensuing scapegoating and othering fueled anxiety and created false binaries, in which, at times, only the ‘others’ were vulnerable to the virus, not ‘us’. Though perhaps inadvertently, ultimately, the compounded effects of the above can be seen almost as promotors of hesitancy and denialism. The field of suspicion in Soweto and Thembelihle was repeatedly amplified to the extent that it eventually swayed the vast majority away from acceptance and into denial. Mistrust in government’s authority and healthcare institutions—alongside the influence of ‘anti-vax’ movements and conspiracy theories propagated on social media—can transform cautious wavering into resolute denial. This then further compromises government’s ability to impart knowledge, build trust, and implement policy. Attention must be given to these phenomena to inform future vaccination programs. Media platforms could be utilized in this process by addressing public concern in a constructive and culturally appropriate manner, particularly in areas where misinformation is rampant. More social behavioral research should be undertaken in different population groups to understand culture-specific issues essential to boosting acceptancy. It is paramount for uptake to comprehend and mitigate social and behavioral circumstances that may impact detrimentally on aspects of acceptability.
